# Electrospun poly (L-lactic acid)/gelatine membranes loaded with doxorubicin for effective suppression of glioblastoma cell growth *in vitro* and *in vivo*

**DOI:** 10.1093/rb/rbab043

**Published:** 2021-07-30

**Authors:** Boxun Liu, Zhizhong Jin, Haiyan Chen, Lun Liang, Yao Li, Guo Wang, Jing Zhang, Tao Xu

**Affiliations:** Tsinghua-Berkeley Shenzhen Institute, Tsinghua University, Shenzhen 518055, China; Department of Neurosurgery, the First Hospital of China Medical University, Shenyang 110122, China; Tsinghua-Berkeley Shenzhen Institute, Tsinghua University, Shenzhen 518055, China; Sun Yat-sen University Cancer Center, State Key Laboratory of Oncology in South China, Guangzhou 510060, China; Tsinghua-Berkeley Shenzhen Institute, Tsinghua University, Shenzhen 518055, China; East China Institute of Digital Medical Engineering, Shangrao 334000, China; Medprin Regenerative Medical Technologies Co., Ltd, Guangzhou 510663, China; Tsinghua-Berkeley Shenzhen Institute, Tsinghua University, Shenzhen 518055, China; Key Laboratory for Advanced Materials Processing Technology, Ministry of Education; Department of Mechanical Engineering, Tsinghua University, Beijing 100084, China; Biomanufacturing and Rapid Forming Technology Key Laboratory of Beijing; Department of Mechanical Engineering, Tsinghua University, Beijing 100084, China

**Keywords:** electrospinning, electrostatic adsorption, glioblastoma, doxorubicin delivery, poly (L-lactic acid)/gelatine

## Abstract

Electrospun membranes are attracting interest as a drug delivery system because of their material composition flexibility and versatile drug loading. In this study, the electrospun membrane was loaded with doxorubicin (DOX) via electrostatic adsorption for long-term drug delivery. DOX loading process was optimized by varying temperature, time, drug concentration, pH and ionic strength of solutions. The loading process did not impair the structural properties of the membrane. Next, we investigated the drug release kinetics using spectroscopic techniques. The composite membranes released 22% of the adsorbed DOX over the first 48 h, followed by a slower and sustained release over 4 weeks. The DOX release was sensitive to acidic solutions that the release rate at pH 6.0 was 1.27 times as that at pH 7.4. The DOX-loaded membranes were found to be cytotoxic to U-87 MG cells *in vitro* that decreased the cell viability from 82.92% to 25.49% from 24 to 72 h of co-incubation. These membranes showed strong efficacy in suppressing tumour growth *in vivo* in glioblastoma-bearing mice that decreased the tumour volume by 77.33% compared with blank membrane-treated group on Day 20. In conclusion, we have developed an effective approach to load DOX within a clinically approved poly (L-lactic acid)/gelatine membrane for local and long-term delivery of DOX for the treatment of glioblastoma.

## Introduction

Glioblastoma is the most common malignant primary brain tumour. Although maximal safe surgical resection is the gold standard treatment, this treatment option is undermined by the growth and infiltration of glioblastoma cells that evade the scalpel [1]. These remaining cells are often managed using chemotherapy. Unfortunately, the delivery of drugs to the brain is hindered by the blood-brain barrier (BBB) and in practice, only a few drug candidates are available for glioblastoma therapy [2]. A promising approach for postoperative control of glioblastoma is to use drug-loaded implants that allow for local delivery of the drug [3]. Membrane-based drug delivery systems, especially those based on electrospun membranes that have high porosity and large specific surface area, show particular promise in this regard [4]. Electrospinning is widely employed in the fabrication of polymer fibres for these drug delivery systems with pore sizes from nanometres to micrometres [5].

In many cases, drugs are loaded onto the polymer fibres of the membrane using chemical bonding [5]. These techniques require complex fabrication processes involving toxic chemicals, and their products may remain in the membrane. In this study, we developed a simple molecular adsorption approach to load drugs to membrane fibres and showed electrostatic interactions within the composite material reduced the rate of drug release, making them suitable for long-term delivery of drugs to treat glioblastoma cells *in vitro* and *in vivo* [6–9].

Doxorubicin (DOX) is a potent cytotoxic chemotherapy drug that has been explored in the treatment of breast, bladder and liver cancers and glioblastoma [10–12]. Free DOX is rarely used to treat brain tumours via systemic administration because the required high concentration leads to off target toxicity. Besides, DOX molecules cannot cross the BBB [13]. To overcome this problem, several groups have developed drug delivery vehicles, including nanoscale liposomes and transplanted polymer scaffolds, to achieve localized delivery of DOX at the site of the tumour [14, 15]. DOX is positively charged at pH 7.0 due to its protonatable amino group, and so it can engage in electrostatic interactions with full or partially negatively charged groups in the drug carrier [16, 17]. For example, DOX has been loaded into negatively charged hyaluronic acid nanoparticles, and in citric acid-stabilized magnetic nanoparticles [8, 16].

The purpose of this study was to study the loading and release of DOX in a clinically approved electrospun membrane (a composite dura substitute), and to investigate the effectiveness of the drug-loaded membranes to suppress the *in vitro* and *in vivo* growth of glioblastoma cells. We developed a simple passive immersion approach to load DOX on poly (L-lactic acid)(PLLA)/gelatine composite electrospun membranes [18, 19]. Gelatine was incorporated into the biomaterial as previous studies have shown it improves biocompatibility and facilitates biodegradation of the composite membranes [20]. First, we investigated the effects of time, drug concentration, temperature, pH and ion strength of the solutions on the efficiency of DOX-loading in membranes. Having established an optimal loading condition, we used a variety of analytical techniques to investigate the structure, mechanical characteristics, surface properties and hydrophobicity of the composite membranes. We also quantified the kinetics of drug release from the membranes *in vitro*. Next, we investigated the *in vitro* cytotoxicity of DOX-loaded membranes using U-87 MG cells and Human Dermal Fibroblasts adult (HDFa) cells. Finally, we investigated the *in vivo* anti-tumour effects of DOX-loaded membranes in nude mice-bearing subcutaneous human glioblastoma xenografts. In summary, our studies demonstrated the effectiveness of DOX-loaded PLLA/gelatine composite electrospun membranes for long term and localized release of DOX to glioblastoma cells, including a pre-clinical study that demonstrated its potential for long-term suppression of the growth of the remaining tumour cells in brain tissue after surgery.

## Materials and methods

### Materials and animals

The PLLA and PLLA/gelatine electrospun membranes used in this study were provided by Medprin (China). The PLLA/gelatine membranes were fabricated with absorbable PLLA (Mn 3.8 × 10^2^ kDa) and gelatine (Type A from porcine skin, 210–250 g Bloom). PLLA and gelatine fibres were deposited in layer-by-layer to form a membrane with a net structure. DOX hydrochloride, temozolomide (TMZ), paclitaxel (PTX) and sodium salicylate (SS) were purchased from Aladdin (Shanghai, China). Gelatine sponge was purchased from Jinling Pharmaceutical Co., Ltd. (Nanjing, China). 3-(4,5-dimethylthiazol-2-yl)-2,5-diphenyltetrazolium bromide (MTT), proteinase K and Triton X-100 were purchased from Sigma-Aldrich Trading Co., Ltd. (Shanghai, China). 4’,6-diamidino-2-phenylindole (DAPI), anti-glial fibrillary acidic protein (GFAP) antibody and anti-Ki-67 monoclonal antibody were provided by Servicebio (Wuhan, China). Human glioblastoma (U-87 MG) cells and HDFa cells were purchased from Cell Bank, Chinese Academy of Sciences (Shanghai, China). Both cells were cultured in Dulbecco’s Modified Eagle Medium with 10% foetal bovine serum, 1% penicillin and 1% streptomycin. Cells were maintained at 37°C in the presence of 5% CO_2_.

Female BALB/c nude mice (4 weeks old) were purchased from HUNAN SJA Laboratory Animal Co., Ltd. (Changsha, China). The mice were fed with sterilized food and water in a sterile environment. All mice were treated in accordance with the guidelines of the Bioethics Committee of Tsinghua Shenzhen International Graduate School (Shenzhen, China), and in compliance with EU Directive 2010/63/EU for animal experiments.

### Drug loading process

DOX was loaded onto membranes using the direct immersion method. Briefly, an aqueous solution of DOX (1 mg/ml) was prepared by dissolving DOX (10 mg) in 10 ml of phosphate buffered saline (PBS, pH 7.4) or ultrapure water and stirring for 1 h at 37°C. Hydrochloric acid (HCl, 2.5% v/v) was used to adjust the pH for studies investigating the effect of pH on DOX loading. The ionic strength of the immersion solution was controlled by adding defined amounts of solid sodium chloride. Electrospun PLLA/gelatine membranes were immersed in a solution containing different DOX concentrations (50, 100, 200 and 500 µg/ml) for defined periods of time (0.5, 1, 2, 6, 18 and 24 h) at 37°C. After the defined time, the membrane was followed by drying in an oven at 37°C. The dried samples were stored in the dark at 4°C for subsequent analyses. The total loaded DOX was calculated based on the difference in DOX content measured by UV-Vis absorbance in the solutions before and after the loading process.

### Characterization of drug-loaded membranes

#### Fluorescence microscope and scanning electron microscopy

The adsorption of DOX in the electrospun membranes was analysed using fluorescence microscopy (Carl Zeiss LSM 700, Thornwood, NY, USA). The morphology and diameter of individual fibres of the electrospun membrane were recorded using scanning electron microscopy (SEM; Thermo Scientific Phenom Pharos, USA) at an accelerating voltage of 10 kV. For the SEM studies, the samples were adhered to an aluminum plate with conductive tape and sputter-coated with gold for 60 s using a coater (Quorum, UK) before imaging.

#### Tensile strength

The tensile strength of the electrospun membranes was recorded by a universal testing machine (Guangdong Kiatest Instruments, China) with a 20 N load cell at a speed of 10 mm/min at 25°C. Dry planar membranes were cut into 20 × 5 mm rectangles and immersed in PBS to wet the membrane prior to the test. Four specimens were tested in each group.

#### Fourier transform infrared and Raman spectroscopy analysis

Infrared spectra analysis of the electrospun membranes was performed using an Fourier transform infrared (FTIR) spectrometer (BRUKER VERTEX 70, Germany) operating over a wavenumber range from 500 to 4000 cm^−1^ with 2 cm^−1^ resolution. Raman spectral analysis of the electrospun membranes was conducted using a Raman spectrometer (HORIBA HR800, USA) using laser excitation at 785 nm.

#### Surface zeta potential measurement

The Zeta potential of the membrane samples was recorded using a SURPASS 3 electrokinetic analyser (Anton Paar, Austria) at 25°C. The samples were cut into 20 × 20 mm squares for the test. The surface zeta potential was measured as a function of pH by varying the solution pH with 0.05 M HCl or 0.05 M sodium hydroxide. The osmolality of the samples was fixed in a 0.1 M potassium chloride electrolyte solution.

#### Water contact angle analysis

The surface water contact angles of the membranes were recorded at 25°C using a contact angle instrument (KSV CAM 200, Finland). The average value of the contact angles of the same sample was reported using three different samples.

### *In vitro* release studies

We recorded the spontaneous release of DOX from PLLA and PLLA/gelatine electrospun membranes in PBS by recording the UV absorbance (480 nm) of DOX in the medium. We also conducted these studies using DOX in gelatine sponges. Proteinase K was used to accelerate the degradation of the electrospun membranes for a better understanding of long-term DOX release [19]. Briefly, the membrane (10 × 10 mm) was soaked in 6 ml of buffer solution (PBS at pH 4, PBS at pH 7.4 or PBS with 50 µg/ml proteinase K at pH 7.4, respectively). The samples in brown bottles were incubated in a shaker (Shanghai Yiheng Scientific Instrument THZ-300C, China) at 80 rpm at 37°C. At selected time intervals, 3 ml of sample solutions were withdrawn and replaced with 3 ml of the same fresh medium. The absorbance of the collected release medium was measured using a UV-Vis spectrophotometer (Thermo Scientific GENESYS 10S, USA) at 480 nm. The amount of DOX released from the membranes was determined by converting the absorbance value to concentration using a calculated extinction coefficient of 9396 M^−1^cm^−1^ for DOX at 480 nm. The calibration curve obtained in the PBS solution is shown as Supplementary Fig. S1.

### *In vitro* cytotoxicity assay

The cytotoxicity of DOX-loaded membranes was evaluated via the MTT assay using U-87 MG and HDFa cells. Cells were seeded at 2 × 10^4^ cells per well in 24-well plates overnight at 37°C. The cell culture medium was replaced with 1 ml medium containing sterilized (gamma irradiation, 40 kGy for 24 h) blank membranes, DOX-loaded membranes, as well as free DOX, and 1 ml fresh medium (negative control). The final concentrations of DOX in DOX-loaded membranes and free DOX were 0.1, 1, 5 or 10 µg/ml. At predetermined time points (24, 48 and 72 h), we removed the culture medium in each well. Next, we added 360 µl of fresh medium and 40 µl of MTT solution (5 mg/ml) into each well. The cells were then incubated for another 4 h at 37°C in the dark. After incubation, we discarded the medium and added 400 µl of dimethyl sulfoxide (DMSO) into each well to solubilize the blue formazan crystals. Three 100 µl aliquots of the supernatant from each well were transferred to individual wells in 96-well microplates, and the absorbance was measured using a microplate reader (BioTek Epoch 2, USA) at a wavelength of 492 nm [21]. For each group, three samples were measured. Untreated cells were served as 100% cell viability. The cell viability of the test groups was calculated using the formula:

$$100\%*(A_{\mathrm{test}}-A_{\mathrm{blank}})/(A_{\mathrm{control}}-A_{\mathrm{blank}}),$$

where $A_{\mathrm{test}}$ represented the absorbance of the test group, $A_{\mathrm{blank}}$ represented the absorbance of DMSO and medium (without cells), $A_{\mathrm{control}}$ represented the absorbance of untreated cells group. 

### *In vivo* anti-tumour evaluation

We employed a U-87 *ex situ* GBM model to evaluate the *in vivo* cytotoxicity of DOX-loaded membranes. Briefly, we injected U-87 MG human glioblastoma cells (2 × 10^6^ cells in 100 µl of PBS) into the right armpits of nude mice (female, 6-week-old). The mice were randomly assigned to two groups (four animals per group). Mice from Group 1 received blank membranes and mice from Group 2 received DOX-loaded membranes. On Day 21 after instilling tumour cells, PLLA/gelatine membranes and DOX-loaded PLLA/gelatine membranes were implanted at the tumour site in the two groups of mice. Following local treatment, the mice were weighed, and the tumours were measured with a Vernier calliper (Meinaite tools, China) every other day. The volume of the tumours was calculated using the following equation: *V* = 0.5236 × length (mm) × width^2^ (mm^2^) [15]. On Day 20 after implanting the membrane, all of the mice were sacrificed, and the tumours were collected for histological analysis. Haematoxylin and eosin (H&E) staining and immunostaining of the paraffin-embedded tissue sections were performed according to standard protocols [22, 23]. The nuclei of tumour cells were stained with DAPI. GFAP antibody was used for the identification of U-87 MG cells. Proliferative tumour cells were detected using an anti-Ki-67 monoclonal antibody.

### Statistical analysis

All experiments were conducted separately, and at least three times. All values are reported as the mean and standard deviation of these independent measurements. Statistical analysis was carried out using one-way analysis of variance and Student’s *t*-test with Origin 2021 software (OriginLab Corporation, Northampton, MA, USA). The criteria for statistical significance were *P <* *0.05, **P <* *0.01 and ***P <* *0.001.

## Results and discussion

### Morphological properties

**Figure 1.**
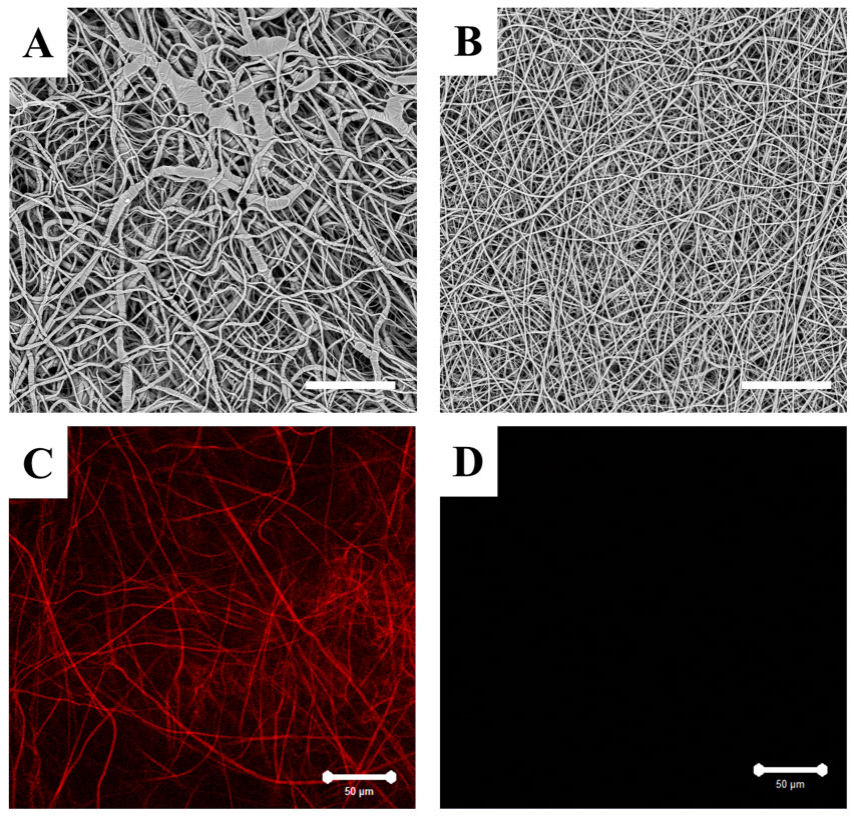
Images of the electrospun membrane. SEM images of (**A**) PLLA membrane and (**B**) PLLA/gelatine membrane. Fluorescence image of (**C**) DOX-loaded PLLA/gelatine membrane and (**D**) blank PLLA/gelatine membrane. The white scale bar represents 50 µm.

Previous studies have shown the introduction of gelatine in PLLA membranes improves the wettability and biocompatibility of the composite membrane [24]. The morphologies of different membranes preparations from this study are shown in Fig. 1A and B. The images showed fibres in the PLLA/gelatine membrane appeared smoother than those of the PLLA membrane, with the average diameter of the former being 799 ± 253 nm compared with 1935 ± 583 nm for the PLLA membrane. When incorporating gelatine into PLLA solution, there has been a marked decline in the value of the solution viscosity (1070 cP for the PLLA solution and 170 cP for the PLLA/gelatine solution). This is likely due to the gelatine with lower molecule weight reduced the chain entanglements of the mixture solution, which is equivalent to decreasing the concentration of PLLA. [25, 26]. We also found that the conductivity of PLLA/gelatine solutions (16.25 µs/cm) was higher than that of PLLA solutions (0.73 µs/cm), which we attribute to the polyelectrolytic feature of gelatine. We postulate the enhanced conductivity of PLLA/gelatine may allow for higher amount of charges carried by the jet that increases the tensile force under the electric field, resulting in thinner fibres [27]. We used fluorescence microscopy to image the intrinsic red fluorescence of DOX and showed DOX was uniformly adsorbed to the nanofibres of the DOX-loaded membrane, whereas fibres without DOX did not exhibit any red fluorescence when excited at 488 nm and observed at 594 nm (Fig. 1C and D).

### Mechanical properties

**Figure 2.**
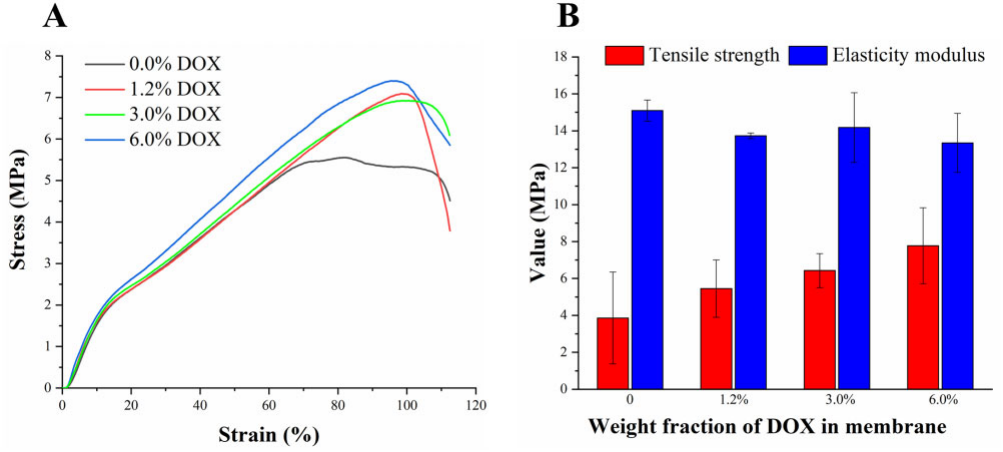
Mechanical characterization of the DOX-loaded PLLA/gelatine membrane. (**A**) Typical tensile stress-strain curves of membranes with different DOX contents. (**B**) Tensile strength and elasticity modulus of membranes with varying DOX contents.

Representative tensile strain-stress curves of the PLLA/gelatine composite membrane loaded with increasing amounts of DOX are shown in Fig. 2A and the tensile strength and elasticity modulus changes are summarized in Fig. 2B. We did not find any significant differences of the elasticity modulus and tensile strength among membranes with different amounts of DOX (*P *>* *0.05). This result is consistent with the largely coincident trend in the linear elastic region of the tensile strain-stress curves. We note PLLA/gelatine membranes have been used to repair the dura following surgery. Previous studies showed that the mechanical strength of the dura substitute should be higher than 3 MPa for preventing cerebrospinal fluid leakage [20, 28]. Significantly, our results show the DOX-loaded membranes fabricated with immersion method can maintain the same mechanical performance of the clinically approved membranes for dura repair. These results demonstrated that the DOX loading onto the membranes via electrostatic adsorption did not alter the structure of the membranes.Cerebrospinal fluid)

### Surface zeta potential

**Figure 3.**
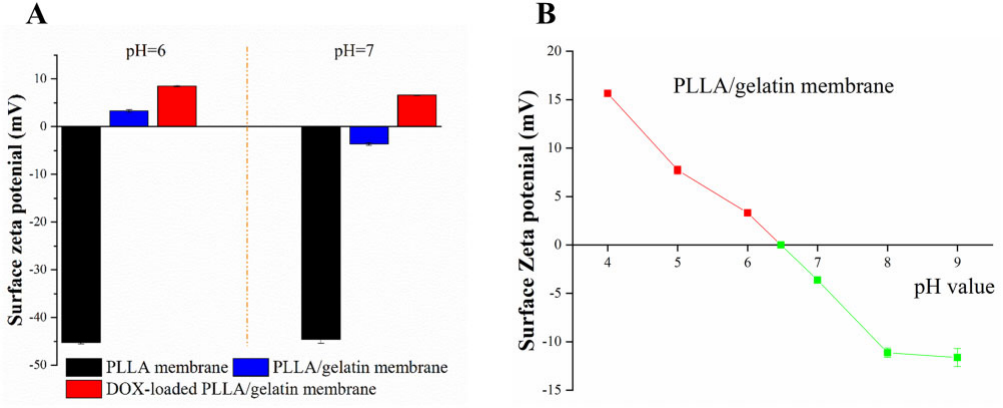
Surface zeta potential of PLLA and PLLA/gelatine membranes under varying pH conditions.

The isoelectric point of gelatine is between pH 6.0 and pH 7.0 [29]. In Fig. 3, we report the surface zeta potentials of electrospun membranes in solutions of various pH. We recorded a uniform value of the zeta potentials of PLLA membrane between pH 6 and pH 7 at approximately −44 mV. After integrating gelatine into PLLA, the zeta potential of the composite membrane was found to be sensitive to the pH, increasing from −3.6 mV at pH 7–3.3 mV at pH 6. This is due to the charge change of gelatine with varying pH. Further, we found that the zeta potential of the membranes varied over a wider range of pH values, ranging from 15.6 mV at pH 4 to −12.5 mV at pH 9 (Fig. 3B). The isoelectric point of the composite membrane was calculated to be at ∼pH 6.5. Since DOX is positively charged at physiological pH (7.0), we focused our passive DOX loading process in solutions at pH values >6.5. After loading DOX onto the membranes, the zeta potential of the DOX-loaded membranes remained positive between pH 6 and pH 7, as shown in Fig. 3A.

### FTIR and Raman spectroscopy

**Figure 4.**
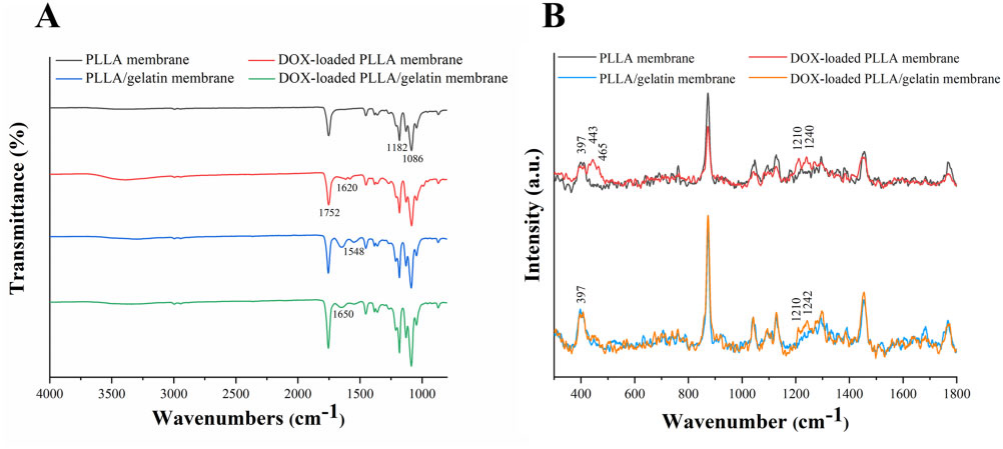
FTIR and Raman spectroscopy. Typical (**A**) FTIR spectra and (**B**) Raman spectra of PLLA, PLLA/gelatine, DOX-loaded PLLA and DOX-loaded PLLA/gelatine membranes.

Next, we investigated properties of PLLA and PLLA/gelatine membranes with and without DOX using FTIR spectroscopy. We recorded several differences in the absorption peaks between these membrane samples, as shown in Fig. 4A. First, we recorded two peaks at 1086 and 1182 cm^−1^ (derived from the C - O-C stretching vibration) in the spectra of all membranes. The absorption peak at 1752 cm^−1^ recorded in both membranes was due to C = O stretching vibrations [30]. We assigned the peaks at 1548 and 1650 cm^−1^ in the spectrum of PLLA/gelatine membrane to the amide II band N-H symmetric deformation absorption peak and amide I band C=O stretching vibration absorption peak of gelatine. This observation reflected the successful incorporation of gelatine into PLLA [31]. The peak recorded at 1620 cm^−1^ in DOX-loaded PLLA membranes is possibly due to the quinone of DOX [32, 33]. We did not find obvious difference between the peaks of PLLA/gelatine membranes and DOX-loaded PLLA/gelatine membranes. This is because that the absorption IR peaks of the PLLA/gelatine membrane are quite intense and may obscure those from DOX [34, 35].

The Raman spectra of PLLA and PLLA/gelatine membranes with or without DOX were recorded using a laser excitation wavelength of 785 nm (Fig. 4B). All the spectra were smoothed and each baseline was subtracted according to the same linear baseline. Each spectrum was normalized by the sum of the Raman intensities. We found one characteristic peak in all spectrums at 397 cm^−1^, which was assigned as CCO deformation of PLA [36]. The peaks recorded at 443 and 465 cm^−1^ were due to δ(C-O) and δ(C=O) plane bending, respectively. The two bands at 1210 and 1242 cm^−1^ (or 1240 cm^−1^) were assigned as ring stretching from C-O [37]. All these peaks were the characteristic features of DOX and were only obtained from DOX-loaded membranes, indicating the successfully adsorption of DOX into both PLLA membranes and PLLA/gelatine membranes.

### Wettability analysis

**Figure 5.**
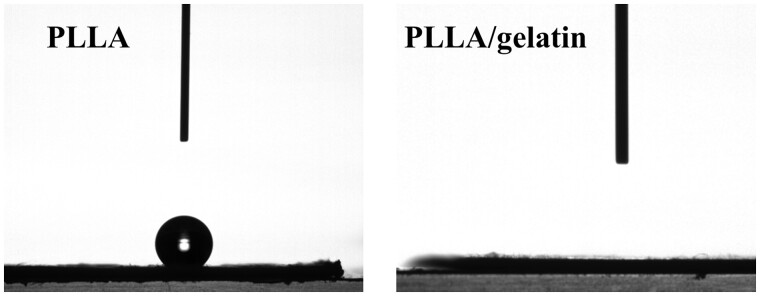
Water droplet image (2 s recording) on PLLA membrane and PLLA/gelatine membrane.

Wettability contributes significantly to the drug-loading capacity of membranes fabricated by the immersion method, and it also influences subsequent drug release. In Fig. 5, we show images of a water droplet on PLLA and PLLA/gelatine membranes. The contact angle of the PLLA membrane was ∼141°, demonstrating the hydrophobicity of this membrane. In contrast, the water droplet on the PLLA/gelatine membrane spread and was adsorbed in <2 s, which would suggest gelatine significantly improved the hydrophilicity of the composite membrane. On the other hand, we needed to pat the droplet on the PLLA membrane to facilitate its entry by capillary action. Thus, the hydrophilic PLLA/gelatine membrane is expected to facilitate the loading of polar drug molecules.

### Drug loading studies

**Figure 6.**
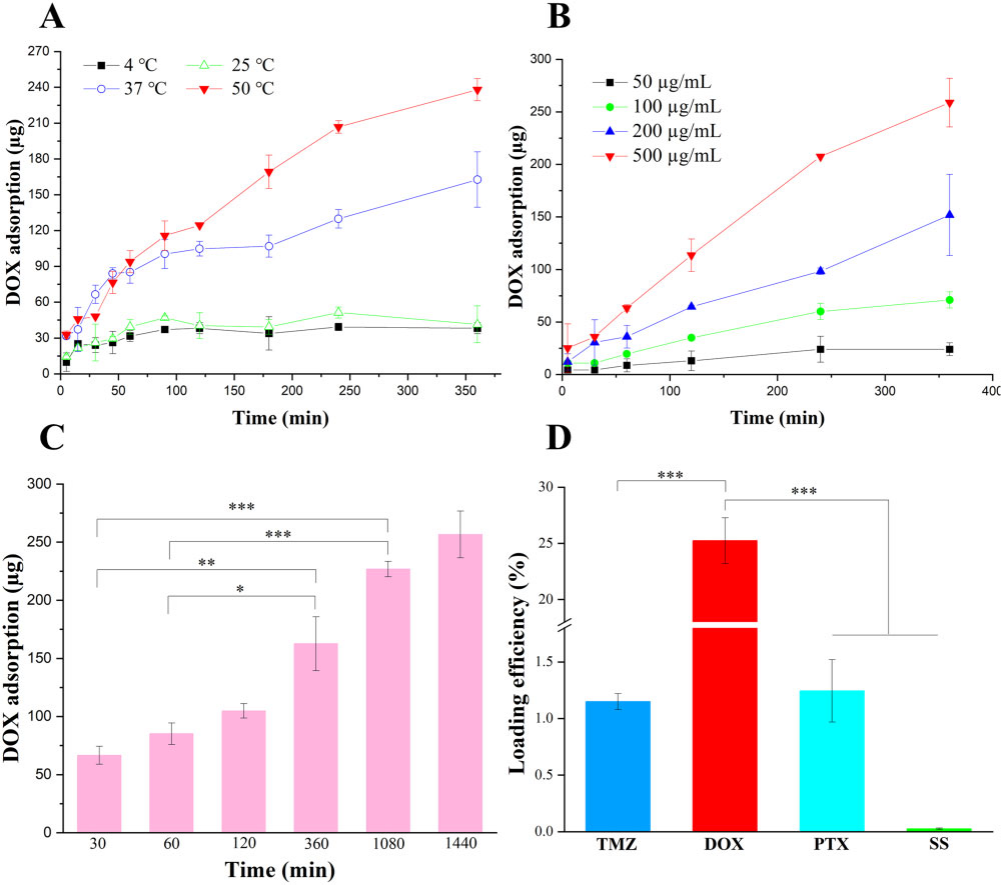
Factors affecting DOX loading of PLLA/gelatine membranes. Influence of (**A**) temperature, (**B**) drug concentration and (**C**) time on DOX loading efficiency. (**D**) Loading efficiency of different drugs on the PLLA/gelatine membranes.

A direct immersion method was used to load DOX onto PLLA/gelatine membranes that were composed of hundreds of layers of negatively charged fibres. We envisage the drug loading process in two steps: first, adsorption of DOX molecules onto the surface of fibres; second, diffusion of DOX molecules into inner layers of the membranes where they bind to negatively charged gelatine molecules. To optimize the loading and retention of DOX in PLLA/gelatine membranes, we conducted studies to show how the loading process was affected by the concentration of DOX, temperature, time, pH and the ionic strength of the solutions. In these studies, we investigated the amount of DOX in uniform samples of the PLLA/gelatine membrane with surface area of 1 × 1 cm^2^ and weight of 5 mg. We found the membranes adsorbed approximately 30 µg DOX over a 6 h immersion period between 4°C and 25°C. At 37°C and 50°C, the membranes absorbed 162 and 238 µg DOX, respectively (Fig. 6A). We attribute this increase to the enhanced movement of the drug molecules caused by higher temperature that promotes diffusion of DOX molecules into the matrix.

The concentration of the drug in the solution is another important factor affecting the efficiency of drug loading in our immersion technique. Diffusion describes the process by which the molecules move from a region of higher concentration to a region of lower concentration. The rate of diffusion is related to the concentration gradient. The loading rate and amount increased with increasing DOX concentration in the immersion solutions (Fig. 6B). At 500 µg/ml DOX, ∼258 µg DOX was adsorbed after 6 h of immersion. We note our membrane exhibits a higher DOX-loading capacity (>50 mg/g) than some other hydrogel-based drug delivery systems (e.g. 22 mg/g) [38]. The time-dependent feature of the DOX adsorption is illustrated in Fig. 6C. As incubation time increased, more DOX was adsorbed by the membrane.

**Figure 7.**
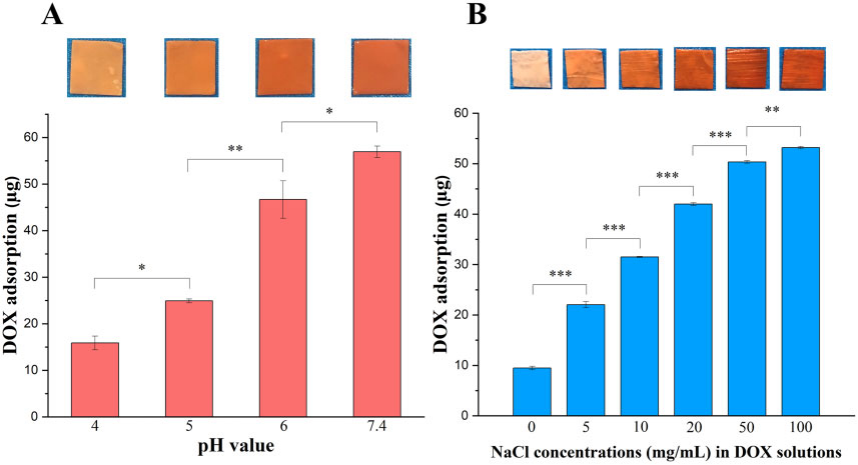
DOX Loading of PLLA/gelatine membranes. DOX adsorption of membranes following immersion into DOX solutions with different (**A**) pH and (**B**) ionic strength for 24 h at 37°C. Images of membranes after immersion into DOX solution with the corresponding conditions are shown at the top of each column.

Drug loading was also shown to be dependent on the pH and the ionic strength of the solution [39]. Figure 7A shows the influence of pH on DOX adsorption. As the pH was lowered, the membranes took up less DOX. As demonstrated by the surface zeta potential analysis described above, the pH affected the surface charge of PLLA/gelatine membranes. At pH values >6.5, the PLLA/gelatine membrane was positively charged. The lower DOX adsorption in acidic solutions is due to the weaker electrostatic interaction between DOX molecules and polymers. Next, we investigated the effect of ionic strength on the drug-loading efficiency. The binding of DOX was markedly enhanced on increasing the NaCl concentration from 0 to 100 mg/ml (Fig. 7B). Although effective, we cannot fully account for the molecular origin of the result as the effect of ionic strength on electrostatic adsorption is complex. One possible explanation for the increase in DOX adsorption is that the high ionic strength decreases the solubility of DOX in the solution thereby promotes DOX precipitation from the solution to the surface of fibres.

Next, we attempted to load PLLA/gelatine membranes with other commonly used drugs like TMZ, PTX and SS. The membranes were immersed into 3 ml drug solutions (0.9% NaCl solutions for TMZ, DOX and SS, ethanol for PTX). We fixed the concentration of each drug at 200 µg/ml and the membranes were immersed for 24 h at 37°C (20°C for TMZ). As shown in Fig. 6D, we found the loading efficiency of DOX (∼25%) was at least 20× higher than the other drugs. As expected, the membranes did not take up the negatively charged SS (<0.03%). The poor loading efficiency of TMZ and PTX (<1.25%) on the membranes was likely attributed to the weak interactions between the electroneutral drug molecules and nanofibres of the membranes. These results further prove the strong efficacy of DOX loading on PLLA/gelatine membranes via electrostatic interaction and demonstrate the potential capacity of these membranes for loading other positively charged drugs. In summary, optimal loading was achieved by immersing the membranes in PBS (pH 7.4) solution at 37°C for 1 day in the dark.

### *In vitro* release studies

Previous studies have shown porous gelatine sponges are useful for haemostasis in neurosurgery and used as a drug carrier of DOX to treat tumours [40–42]. In our studies, we compared gelatine sponge, PLLA membrane and PLLA/gelatine membrane in a quantitative DOX release study.

**Figure 8.**
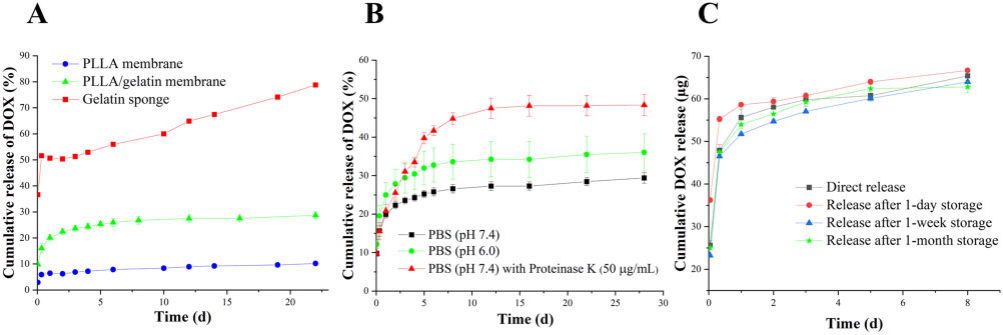
*In vitro* DOX release profile. (**A**) DOX release from PLLA membrane, PLLA/gelatine membrane and gelatine sponge in PBS. (**B**) DOX release from PLLA/gelatine membrane in PBS (pH 7.4), PBS (pH 6.0) and PBS (pH 7.4) with proteinase K (50 µg/ml). (**C**) DOX release after specified storage times.

The gelatine sponge is hydrophilic and highly absorbent, having a swelling ratio of more than 900% [43]. These features facilitate its drug loading during water absorption. The great hydrophilicity and porosity of the gelatine sponge on the other hand could result in the rapid release of DOX. In kinetic studies of DOX release from pure gelatine sponges (Fig. 8A), we found that DOX was indeed released rapidly from the gelatine sponge that exhibited a burst release of ∼36.6% in the first 1 h. It was followed by a slower but still unacceptable rapid rate of release. Although the burst release of DOX would trigger to a rapid cytotoxic effect on the tumour, it could also lead to cytotoxic side effects on normal cells. From the kinetic data, it is also evident that the gelatine sponge released most of the DOX within 24 h, and so it is not useful for sustained delivery of DOX. As we discussed earlier, the PLLA membrane alone does not take up DOX very efficiently. We also found that the entrapped drug molecules were released very slowly (<10% in 3s), making it an unsuitable vehicle for drug delivery. Therefore, both the gelatine sponge and the PLLA membrane do not meet the criteria we set out for sustained delivery of drugs to solid tumours.

The PLLA/gelatine membrane demonstrated a two-stage release behaviour: an initial burst release that ∼22% of DOX was released in the first 2 days, followed by a slow sustained release over more than 3s (Fig. 8A). The burst release involved DOX molecules distributed on the outermost layer of the fibres which perhaps was deposited via a precipitation process. An acceptable initial burst drug release is beneficial to killing tumour cells, contributing to a strong therapeutic effect at the beginning of the treatment. The subsequent sustained release could maintain an effective concentration of drugs over an extended period, reducing the risk of recurrence of local tumours [44].

In previous studies, DOX has been incorporated into the PLLA-based fibres via emulsion-electrospinning or blending electrospinning [44, 45]. In those delivery systems, DOX molecules were distributed in the centre of the nanofibres, followed by the diffusion-controlled release. The release periods for 50% DOX in those studies were between 5 and 15 h, which were too fast for clinical use. In our systems, more than 70% of DOX were still on the membranes after 4 weeks’ release *in vitro*. The long-term release potency of these membranes is attributed to the strong electrostatic interaction between DOX molecules and fibres. In addition, the physical adsorption for DOX loading did not impair the structure of the membranes, which also contributes to the sustained DOX release during the slow degradation of the membranes. Our systems were expected to achieve better performance for long-term inhibition of tumour growth due to the sustained DOX release.

DOX release in pH 6.0 PBS solution was faster than that in pH 7.4 PBS. At pH 6.0, 34% of DOX was released from the PLLA/gelatine membrane in 16 days. In contrast, only 27% of DOX was released at pH 7.4 over the same time period (Fig. 8B). The average DOX release rate within 16 days at pH 6.0 was 1.27 times as that at pH 7.4. This is because that lower pH leads to protonation of carboxylic groups in the polymers and their negative charges will be neutralized [46]. In this case, the electrostatic interaction between the drug molecules and polymers will be weaker, thereby facilitating the DOX release. The pH-dependent DOX release behaviour of the DOX-loaded membrane can contribute to an enhanced anti-tumour effect in a mildly acidic tumour microenvironment [47].

The degradation of PLLA is generally slow both *in vitro* and *in vivo* because of its high crystallinity and hydrophobicity [48]. Previous studies have shown that pure PLLA has little mass loss for at least 4 weeks [49]. Although gelatine promoted the mass loss of the PLLA/gelatine membrane to 17% after 4 weeks, it was not convenient to study the overall drug release. Proteinase K was used to accelerate the degradation of the PLLA/gelatine membrane, and this made it possible to study the overall drug release profiles in a short observation time period [50]. There was a significant acceleration of DOX release in PBS with proteinase K compared with that in PBS only (Fig. 8B). Nearly 46% of DOX was released with the membrane broken into short fibre fragments. Degradation of the fibres increased the contact area between DOX and the solution, enhancing the desorption process. Nonetheless, over 50% of the DOX was still bound on the fibres after 3 weeks, holding the potential for long-term release.

The stability of DOX on the membrane was also investigated by measuring the release profile after different storage times (Fig. 8C). The DOX-loaded membrane was prepared and stored at 4°C in a dry state. As shown in Fig. 8C, the membranes exhibited similar release features both in wet state (direct release) and dry state (release following a specified storage time). The storage time (from 1 day to 1 month) had no obvious influence on the release profile, demonstrating that DOX was stable on the membrane for more than 1 month. The observed DOX stability should facilitate scale-up manufacturing for clinical use.

### *In vitro* cytotoxicity

**Figure 9.**
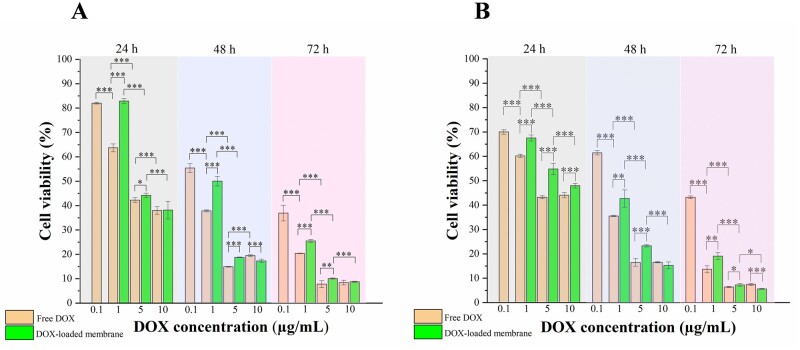
*In vitro* cytotoxicity. Cell viability of (**A**) U-87 MG cells and (**B**) HDFa cells incubated with free DOX and DOX-loaded PLLA/gelatine membranes measured at 24, 48 and 72 h at the indicated drug concentrations.

To evaluate the *in vitro* anti-tumour effects of DOX-loaded PLLA/gelatine membranes, U-87 MG cells were incubated with these membranes and the cell survival rate was measured using the MTT assay (Fig. 9). In addition, HDFa cells were incubated with the membranes to evaluate the influence of drug-loaded membranes on wound repair. U-87 MG cells and HDFa cells were treated with different samples for 24, 48 and 72 h. Free DOX was used as a positive control, and the medium only was used as a negative control. As shown in Fig. 9A, the cytotoxic effects of both free DOX and DOX-loaded membranes on U-87 MG cells enhanced with an increase in total DOX concentration and incubation time. When the DOX concentration was 1 or 5 µg/ml, The DOX-loaded PLLA/gelatine membranes showed a significantly lower inhibition effect than free DOX in the 24–72 h period. This was because most DOX molecules were still entrapped by the membrane during the first 3 days thus producing a lower DOX concentration in the medium than that of free DOX. However, when the DOX concentration was increased to 10 µg/ml, no significant difference between the DOX-loaded membrane groups and free DOX groups was observed. This can be explained that membranes loaded with DOX at concentration of 10 µg/ml could release enough DOX to trigger equivalent damage to tumour cells within 1 day compared with free DOX. A similar cytotoxic effect of the DOX-loaded membranes on HDFa cells suggested that the DOX-loaded membrane could not be implanted into a wound site (such as the dura damage site) when conducting local chemotherapy, as there was a risk of delayed wound healing. In conclusion, DOX-loaded membranes with sustained DOX release can be expected to achieve long-term inhibition of cancer cells growth once the membrane is implanted at a tumour site.

### *In vivo* studies

**Figure 10.**
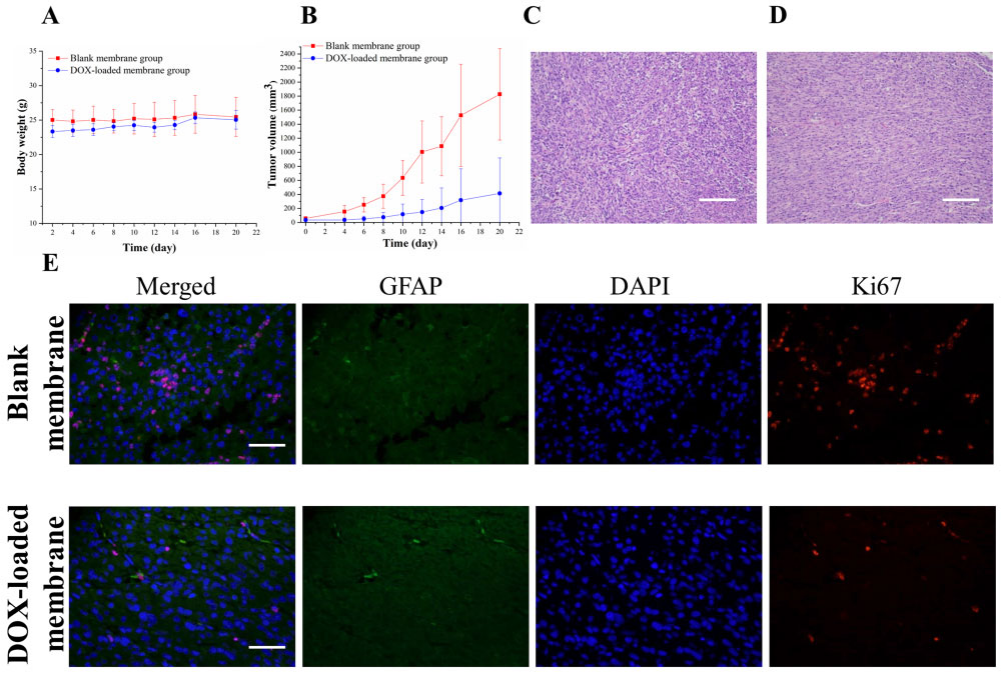
*In vivo* anti-tumour activity of DOX-loaded PLLA/gelatine membrane in nude mice-bearing subcutaneous glioblastoma xenografts. (**A**) Change in body weight of nude mice. (**B**) Change in volume of tumours. H&E staining of tumour sections from (**C**) Blank membrane-treated group and (**D**) DOX-loaded membrane-treated group. The scale bar is 200 µm. (**E**) Immunofluorescence staining of tumours. Green, blue and red fluorescence represent GFAP-stained cell, DAPI-stained cell nuclei and Ki-67-stained cell nuclei, respectively. The scale bar is 50 µm.

We conclude from above kinetic and *in vitro* cytotoxicity studies that the membranes allow for long-term delivery of DOX at a concentration that may be beneficial to the suppression of tumour cell growth *in vivo*. The *in vivo* anti-tumour efficiency of DOX-loaded PLLA/gelatine membranes were evaluated in nude mice-bearing subcutaneous glioblastoma xenografts. In all groups (with or without DOX), the mice maintained their body weight over 3 weeks, indicating that there was no obvious toxicity in the local chemotherapy with the DOX-loaded membrane (Fig. 10A). As shown in Fig. 10B, tumour growth was significantly inhibited in the DOX-loaded membrane-treated groups in comparison with that in the blank membrane-treated groups (*P* < 0.001). After treatment with blank membrane and DOX-loaded membrane, mean tumour size at Day 20 was 1826.28 ± 651.18 and 414.09 ± 506.01 mm^3^, respectively.

H&E staining and immunofluorescence staining (antibodies against GFAP and Ki-67) were performed on the two treatment groups to further evaluate the therapeutic effects of DOX-loaded membrane. Sections obtained from the blank membrane group showed higher cell density and darker staining compared with that from the DOX-loaded membrane group (Fig. 10C and D). GFAP is highly expressed in glioblastoma as a cytoskeletal protein. Both groups exhibited GFAP expression, and no significant difference was found between the two groups (Fig. 10E). Ki-67 is a nuclear antigen absent in resting cells that is used as a marker for proliferating cells. The Ki-67 labelling signal reflects the percentage of positively stained proliferating cells over total cells. According to the Ki-67 staining assay, the DOX-loaded membrane treatment led to less cell proliferation compared with the blank membrane treatment, further demonstrating the anti-tumour efficacy of the DOX-loaded membrane (Fig. 10E). Taken together, the results suggest that DOX released from the implanted PLLA/gelatine membrane effectively inhibited tumour cell proliferation, resulting in reduced tumour growth in glioblastoma-bearing mice.

In summary, we have developed an effective and facile approach to load DOX onto a clinically approved PLLA/gelatine membrane for the local treatment of gliomas. When compared with the *in situ* gel systems, the biosafety concern of the developed membranes was minimized by employing clinically approved biomaterials including PLLA and gelatine incorporated with commonly used drugs. We can avoid the risk of photoinitiator-, UV light- and free radical-induced cytotoxicity which frequently occur in *in situ* gel systems [51–55]. The developed DOX-loaded membranes can be prepared in advance, and are easily attached to the border of the cavity following surgical resection of the tumour. The released DOX can interact with the remaining tumour cells directly, without complicating the operation and causing secondary damage. In consideration of the verified biosafety, high feasibility and effective *in vitro* and *in vivo* anti-tumour activities of the DOX-loaded membranes, we expect that these membranes are much closer to clinical practice compared with those *in situ* gel systems.

## Conclusion

In this work, DOX was loaded onto a clinically approved electrospun membrane via electrostatic adsorption for the local treatment of gliomas. The DOX loading process of PLLA/gelatine membranes was regulated by water absorption and electrostatic interactions between the charged drug molecules and nanofibres, without altering the uniform and bead-free fibrous structure and impairing the mechanical strength of the membranes. These composite membranes also have the potential to serve as carriers for other positively charged drugs. The DOX-loaded membranes exhibited a burst release of DOX reaching 22% in the first 2 days followed by a sustained drug release profile over 4 weeks. In addition, these membranes exhibited a pH-response release that achieved a faster DOX release at pH 6.0 than that at pH 7.4, demonstrating their suitability in the acidic tumour environment. The developed membranes showed effective inhibition of tumour cell proliferation and tumour growth both *in vitro* and *in vivo*. DOX-loaded membranes transplanted in nude mice-bearing subcutaneous glioblastoma xenografts were found to reduce tumour volume by 77.33% as compared with the blank membrane treatment on Day 20. These membranes hold immense potential for clinical use due to their simple material combination of clinically approved biomaterials and commonly used drugs, the feasibility of adhering to the surgical resection margin and effective inhibition of tumour growth.

## Supplementary data

Supplementary data are available at *REGBIO* online.

## Supplementary Material

rbab043_Supplementary_Data
