# Supplementary material for: Electrospun poly (L-lactic acid)/gelatine membranes loaded with doxorubicin for effective suppression of glioblastoma cell growth in vitro and in vivo
Source: Regen Biomater. 2021 Jul 30;8(5):rbab043. doi: 10.1093/rb/rbab043 (PMC8358479; doi:10.1093/rb/rbab043)
Supplement: rbab043_Supplementary_Data [file rbab043_supplementary_data.docx]

**Supplementary Information**

**Electrospun Poly (L-lactic acid)/gelatin membranes loaded with doxorubicin for effective suppression of glioblastoma cell growth *in vitro* and *in vivo***


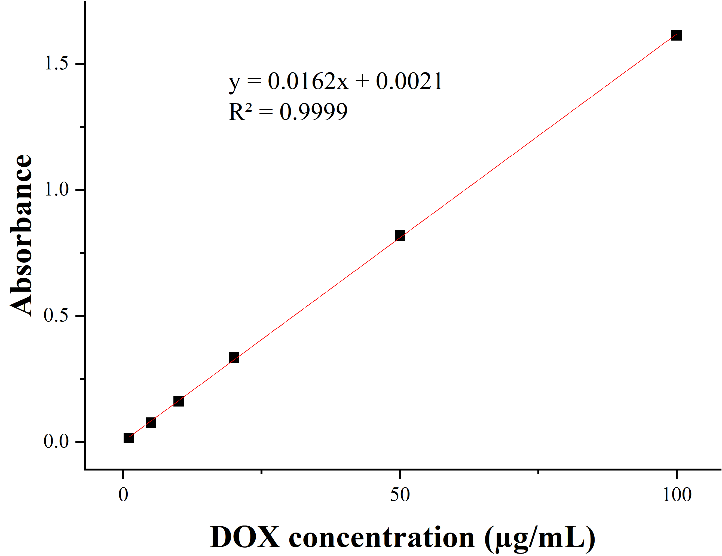


**Fig. S1. UV-vis calibration curve (480 nm) of free DOX in PBS solution**
